# Miniature integrated spectrometers towards high-performance and cost-effective

**DOI:** 10.1038/s41377-023-01302-3

**Published:** 2023-10-30

**Authors:** Haoxuan Sun, Yicheng Zhou, Liang Li

**Affiliations:** School of Physical Science and Technology, Jiangsu Key Laboratory of Thin Films, Center for Energy Conversion Materials & Physics (CECMP), Soochow University, Suzhou, 215006 China

**Keywords:** Optoelectronic devices and components, Optical sensors

## Abstract

The conjugated mode of bound states in a continuum is integrated as a narrowband wavelength extraction unit. A low-cost and easy-to-prepare strategy, using solution-processable semiconductors, has been demonstrated to form a new platform for on-chip spectral analysis.

A spectrometer is an optical instrument that can identify substances based on their response to the wavelength of light, revealing useful information about the properties and composition of light sources and materials^1^. It is extensively used in various fields and applications, such as medical diagnosis^2^, environmental monitoring^3^, chemical analysis^4^, and materials science^5^. Traditional spectrometers often rely on mechanical and optical components for spectral separation and detection. However, such components are large, bulky, expensive, poorly efficient, and difficult to calibrate and thus limit the performance and application of spectrometers for fast, convenient, and efficient spectral measurements^6–10^. Therefore, developing a new type of miniaturized, low-cost, and integrated spectrometer is necessary and urgent.

To miniaturize spectrometers, mechanical structures in traditional spectrometers should be removed first. As such, the miniaturized spectrometers reported to date primarily adopt two strategies: (i) rational design of optical structures (such as dispersive optics^11,12^, metasurface^13–15^, and waveguide^16,17^) and (ii) spatial or temporal spectral-response modulation. The spectral detection performance of miniaturized spectrometers constructed using the former strategy is comparable with that of traditional desktop spectrometers. For example, Wang et al. reported a metal metasurface–material-based metal lens spectrometer that uses wavelength and phase multiplexing to accurately map wavelength information to focal points on the same plane, thereby achieving a spectral resolution of 1 nm^18^. This compact and ultrathin metal lens spectrometer can be applied in on-chip integrated photonics for spectral analysis and information processing on compact platforms. Yao et al. devised a reconfigurable photonics–based on-chip spectrometer^19^ that uses distributed filters to generate ultrawideband pseudorandom spectral responses, effectively reducing the number of sampling channels required while preserving the spectral information. This on-chip spectrometer leverages the advantages of reconfigurable photonic spectral shaping to select and modulate different spectral signals in different wavelength ranges using the custom-engineered distributed filters embedded in its structure. Notably, its spectral resolution can reach the picometer level. However, this strategy entails several issues, including complex fabrication processes and high costs. The spectral-response modulation mechanism can greatly simplify the device structure and reduce the associated costs. Furthermore, the device does not require additional optical and mechanical structures, and even two-dimensional (2D) or low-cost perovskite materials can be used to realize spectral analysis via single detector. Deng et al. and Yoon et al. demonstrated the feasibility of applying external electric fields to induce interlayer optical transitions in 2D heterostructures for spectral analyses using a single detector^20,21^. Guo et al. further modulated the spectral response of organic–inorganic hybrid perovskite devices in the visible-light wavelength range by varying external electric fields in situ^22^. Unfraternally, these spectrometer designs are limited in spectral richness, sensitivity and detectable range, require reconstruction algorithms with oversampling, which compromises the spectral-response immediacy, and perform below the benchtop spectrometer level. Therefore, the low-cost fabrication of on-chip high-performance spectrometers remains challenging.

Recently, Li et al. published a paper in *Light: Science & Applications* that proposed a solution-processable semiconductor-based integrated spectrometer platform that uses a novel photonic phenomenon, namely, conjugated bound states in the continuum (conjugated-BIC), as illustrated in Fig. 1^23^. This design balances high-performance and low-cost manufacturing of an on-chip spectrometer by integrating low-cost solution-processable perovskite materials using a planar waveguide and periodic grating structure. This photodiode can detect light at a specific wavelength, while completely rejecting light at other wavelengths, with remarkable sensitivity. It has a high spectral resolution of up to 3.6 nm and a wide spectral bandwidth of 400–700 nm, which can be flexibly adjusted by changing the grating structure parameters. Furthermore, the compact spectral measurement system of this photodiode seamlessly integrates with other miniaturized devices on the same chip. Li et al. also arranged these ultranarrowband photodiodes in an array to construct an integrated spectrometer platform based on solution-processable semiconductors. This platform can be easily developed using low-temperature solution-processed semiconductor thin films and a single-step-etched grating structure. The integrated device can be used in narrowband/broadband light reconstruction, in situ hyperspectral imaging, and other diverse applications, such as point-of-care diagnosis and IoT-based sensing-and-chip laboratory platforms. This strategy provides guidance for the development of next-generation miniaturized integrated spectrometers.

**Fig. 1:**
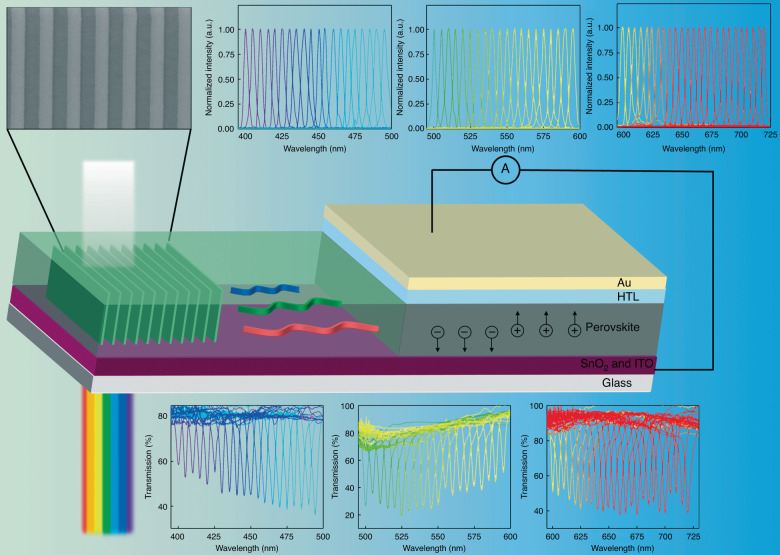
Schematic of the conjugated-BIC photonic spectrometer operational principle. The spectrometer integrates conjugated-BIC photonics and waveguides using perovskite photodiodes. Ultranarrowband responses of the photodiodes can be obtained by customizing the grating structure

Looking forward, the common goal of optical structure design and spatial/temporal spectral-response modulation strategies is to develop miniaturized spectrometers with excellent performance, low cost, and easy processability. The former should focus on reducing the cost, manufacturing complexities, and volumes of the optical structure volume and the entire detection system. By contrast, the response modulation approaches should focus on the robustness of emerging materials (black phosphorus^24^, ionic semiconductors^22^, etc.) based devices, realizing controlled large-scale fabrication^20,21^, and broadening the operational spectral range^6^. Moreover, additional power consumption, heating, and an overload of the computing power of the embedded system owing to complex reconstruction algorithms should also be mitigated.
